# Risk stratification of residual abscess after surgical treatment for gastroduodenal perforation

**DOI:** 10.1002/ags3.12877

**Published:** 2024-11-04

**Authors:** Kana Ishikawa, Siyuan Yao, Takashi Kumode, Keisuke Tanino, Yugo Matsui, Shusaku Honma, Shinichi Hosokawa, Teppei Murakami, Takatsugu Kan, Sanae Nakajima

**Affiliations:** ^1^ Department of Surgery Kobe City Medical Center West Hospital Kobe Hyogo Japan; ^2^ Department of Surgery, Division of Liver and Pancreas Transplantation, The Dumont‐UCLA Transplantation Center David Geffen School of Medicine at UCLA Los Angeles California USA

**Keywords:** ascites culture, chemotherapy, gastroduodenal perforation, NSAIDs, renal function, residual abscess

## Abstract

**Aims:**

Residual abscess is a major complication after emergency surgery for gastroduodenal (GD) perforation. However, there is little evidence regarding potential risk factors contributing to its development. Establishing a risk stratification strategy would be valuable for the entire management process.

**Methods:**

This single‐center, retrospective study analyzed 115 consecutive patients who underwent surgery for GD perforation between 2010 and 2023 at a secondary emergency care hospital. Patients were divided into two groups based on the presence or absence of residual abscesses. Potential risk factors for abscess formation were evaluated from various aspects.

**Results:**

The incidence of residual abscesses was 19.1% (22 of 115). Multivariable analysis revealed that current use of nonsteroidal antiinflammatory drugs (odds ratio [OR] 3.76, *p* = 0.037), cancer chemotherapy (OR 13.56, *p* = 0.005), and preoperative renal dysfunction (OR 4.72, *p* = 0.018) were independent predictors. A potential scoring model could be created using these three parameters, and the number of risk factors correlated with the likelihood of developing a residual abscess (0 vs. 1 vs. ≥2; 6.2% vs. 29.4% vs. 50.0%, *p* < 0.001). From a bacteriological point of view, the presence of *Enterococcus* in the ascites culture was closely related to its occurrence with 100% probability. Moreover, regarding early detection of this complication, C‐reactive protein on postoperative d 5 had the highest predictive ability with an area under the curve of 0.818.

**Conclusion:**

The risk of residual abscess formation after surgical treatment of GD perforation can be assessed utilizing both preoperative and postoperative information.

## Graphical abstract

This report highlights the optimal risk stratification strategy for residual abscesses after surgical gastroduodenal (GD) perforations treatment. Since the pathogenesis of residual abscess is likely to be multifactorial, careful evaluation of potential risk factors from all angles will help us to provide prompt and precise treatment to patients, including medication history, renal function, microorganisms, and inflammatory biomarkers.

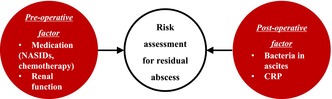

## 1 INTRODUCTION

Gastroduodenal (GD) perforation is a common and highly critical emergency in abdominal surgery. Its morbidity and mortality have been reported to be 30%–70% and 10%–30%, respectively.^1^ Residual abscess, a type of organ/space surgical site infection, is a major complication after emergency surgery for GD perforation. In a recent study using a nationwide surgical registration system in Japan, the frequency was reported to be 8.0% based on data from 2016 to 2019.^2^

The etiology of residual abscess is multifactorial, with delayed wound healing and severe preoperative contamination thought to be the leading causes. However, there is little evidence regarding potential risk factors that specifically contribute to their formation, whereas preoperative general condition, anemia, and renal function have been reported as risks for overall morbidity after surgery,^1^ and pelvic ascites on computed tomography (CT) and sequential organ failure assessment score on admission have been identified as independent factors for failure of nonsurgical management.^3^ The entire treatment process would be more efficient if clinical patient information could be used to predict postoperative abscess formation.

Furthermore, the optimal time to perform tests to assess residual abscesses is still being determined, while early detection is of utmost importance. We hypothesize that postoperative information such as ascites culture results and inflammatory markers should also be carefully evaluated to provide seamless care to patients.

To address these clinical issues, a retrospective study was conducted to identify predictors of postoperative residual abscess formation from multiple perspectives.

## 2 PATIENTS AND METHODS

### 2.1 Study population

**FIGURE 1:**
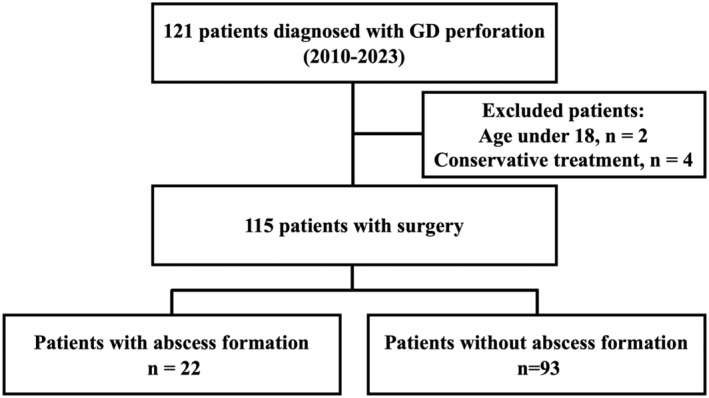
Patient population flow diagram.

The study population is shown in Figure 1. This was a single‐center retrospective observational study of patients diagnosed with GD perforation who underwent emergency surgery at Kobe City Medical Center West Hospital between 2010 and 2023. Among 121 consecutive cases, 115 were included after excluding the following cases: four with nonoperative management and two with age under 18. All patients were Asian.

### 2.2 Study design

Patients were assigned to one of two groups: Abscess group or No‐abscess group. First, the baseline characteristics and perioperative outcomes were compared between the groups. Second, a risk factor assessment for the residual abscess was performed and a scoring model was created using the identified predictors. Third, another potential risk factor was assessed from the perspective of microorganisms isolated from ascites cultures. Finally, since the timing of diagnosis is important in the clinical setting, we tried to find out which postoperative inflammatory parameter was the most powerful predictor. The follow‐up period was from diagnosis to the first postdischarge visit.

The protocol for this research project was approved by the Kobe City Medical Center West Hospital Ethics Board (Approval no. 23‐023) and conforms to the Declaration of Helsinki 2013 provisions.

### 2.3 Surgical indication and procedure

The diagnosis of GD perforation was based on computed tomography (CT) scans. Emergency surgery was generally performed, except in the following conditions: young patients, cases with free air in the retroperitoneum, refusal of surgery, and terminal stage of cancer. However, the surgery was performed even though the cancer was stage IV if the patient had maintained performance status or they demanded it.

The choice of surgical approach was at the surgeon's discretion. Each operator selected the open or laparoscopic approach and omental plugging (Cellan–Jones repair) or omentopexy (primary suture reinforced by omental overlay). If the perforation site was not visible, lavage with drain placement was performed. After repair of the perforation site, the peritoneal cavity was thoroughly irrigated with normal saline until the fluid was clear. Drains were placed to drain any remaining fluid (up to 4 sites: near the perforation site, right subphrenic space, left subphrenic space, and pelvis).

### 2.4 Perioperative management

A nasogastric tube (NGT) was inserted for decompression, and therapeutic antibiotics and a proton pump inhibitor (PPI) were administered intravenously immediately after diagnosis. Generally, a broad‐spectrum antibiotic, such as cefmetazole, tazobactam/piperacillin, or ampicillin/sulbactam was selected, while antifungals were not routinely used. After surgery, all patients were maintained on broad‐spectrum antibiotics and PPIs. The NGT was removed when output was less than 100 mL, and patients were started on a total liquid diet with NGT removal. Antibiotic de‐escalation was based on the results of ascites culture and susceptibility testing. The median duration of the first antibiotic administration was 6 days, and the median number of days to start solid food intake was 5 d postoperatively.

### 2.5 Definitions

The estimated glomerular filtration rate (eGFR) was calculated using the formula published by the Japanese Society of Nephrology.^4^ Since the median age in this cohort was 68 y old, a certain percentage of patients might have an age‐appropriate decline in renal function before the onset of GD perforations. To focus more on functional decline associated with inflammation and dehydration caused by peritonitis, this study defined moderate grade (eGFR <50 mL/min/1.73m^2^) as renal dysfunction.

Postoperative complications were defined according to the Clavien–Dindo (CD) classification.^5^ The diagnosis of residual abscess was based on the CT findings, which were accompanied by clinical symptoms (fever and tenderness) and abnormal blood tests (increased inflammation). CT scans were reviewed by at least two physicians, including an attending surgeon and radiologist (residents and fellows are not allowed to make a diagnosis independently).

### 2.6 Statistical analysis

Continuous variables are presented as the median and interquartile range. Categorical variables are presented as numbers and percentages. Comparisons were performed using the Mann–Whitney *U* test for continuous variables and Fisher's exact test for categorical variables, as appropriate. Logistic regression analysis was used to identify risk factors and their odds ratio (OR) and 95% confidence interval (CI). Factors with a *p‐*value <0.10 in the univariable analysis were subsequently used in the multivariable analysis. Receiver operating characteristic (ROC) analysis was used to compare the predictive ability of each variable for abscess formation. A *p*‐value less than 0.05 was considered statistically significant. JMP 17.0 (SAS Institute, Cary, NC, USA) was used for all statistical analyses.

## 3 RESULTS

A total of 69 men (60%) and 46 women (40%) with a median age of 68 y (range 23–96) were included. The stomach (32.2%) and duodenum (67.8%) were perforation sites. Malignancy was identified as the cause in 10 patients (8.7%), with nine gastric cancers and one metastatic cancer. Laparoscopic procedures were performed in 103 patients (89.6%), and reoperation was required in four (3.5%). In‐hospital mortality within 90 d was observed in four patients (3.5%); two due to the progression of stage IV gastric cancer (one case with intestinal ischemia caused by severe dehydration and another with tumor hemorrhage and cachexia), one due to infection, and one due to nonocclusive mesenteric ischemia.

The selected antibiotics on admission were as follows: cefmetazole (60.0%), tazobactam/piperacillin (15.7%), ampicillin/sulbactam (12.2%), cefazolin (7.0%), meropenem (2.6%), and other (2.6%).

### 3.1 Baseline characteristics

A total of 22 patients (19.1%) developed a postoperative residual abscess. The required treatment was antibiotics alone in 13 patients (59.1%), percutaneous drainage in five patients (22.7%), and reoperation in four patients (18.2%). Major leakage with poor response to conservative treatment was the reason in three cases, and all of them had advanced age ≥83 y old. Abscess accompanied by major wound dehiscence was the reason for another case. Clinical characteristics and postoperative outcomes were compared between groups (Table 1).

**TABLE 1:** Clinical characteristics.

Variables	Total (*n* = 115)	Abscess (*n* = 22)	No‐abscess (*n* = 93)	*p* value
Age, y	68 (23–96)	72.5 (56–96)	66.0 (23–95)	0.003
Gender, female	46 (40.0%)	13 (59.1%)	33 (35.5%)	0.044
Comorbidity				
Diabetes mellitus^a^	14 (12.2%)	6 (27.3%)	8 (8.6%)	0.027
Regular use of NSAIDs	28 (24.4%)	10 (45.5%)	18 (19.4%)	0.015
Cancer chemotherapy^a^	7 (6.1%)	4 (18.2%)	3 (3.2%)	0.024
Perforation site				0.643
Stomach	37 (32.2%)	8 (36.4%)	29 (31.2%)	
Duodenum	78 (67.8%)	14 (63.6%)	64 (68.8%)	
Cause of perforation^a^				0.401
Cancer	10 (8.7%)	3 (13.6%)	7 (7.5%)	
Benign ulcer	105 (91.3%)	19 (86.4%)	86 (92.5%)	
ASA‐PS ≥3	24 (20.9%)	8 (36.4%)	16 (17.2%)	0.047
Time from onset to surgery ≥72 h^a^	9 (7.8%)	4 (18.2%)	5 (5.4%)	0.067
WBC, /μL	10 950 (990–28 330)	10 075 (990–25 030)	11 140 (1200–28 330)	0.280
eGFR, mL/min/1.73m^2^	72.0 (3.5–155.2)	45.7 (9.6–84.8)	82.9 (3.5–155.2)	<0.001
CRP, mg/dL	2.6 (0.0–42.8)	6.0 (0.0–41.6)	1.6 (0.0–42.8)	0.013
Surgical approach				0.459
Open	12 (10.4%)	3 (13.6%)	9 (9.7%)	
Laparoscopy	103 (89.6%)	19 (86.4%)	84 (90.3%)	
Procedure				0.290
Omentopexy	69 (60.0%)	16 (68.2%)	53 (58.1%)	
Omental plug	39 (33.9%)	6 (27.3%)	33 (35.5%)	
Drainage	5 (4.4%)	0 (0%)	5 (5.4%)	
Primary suture	2 (1.7%)	1 (4.6%)	1 (1.1%)	
Operation time, min	90 (34–276)	97 (69–241)	84 (34–276)	0.058
Amount of bleeding, mL	0 (0–350)	0 (0–350)	0 (0–250)	0.220
Irrigation volume, L^b^	6 (1–15)	6 (1–10)	6 (1–15)	0.770
Solid food intake, d	5 (1–21)	7 (2–21)	4 (1–20)	<0.001
Duration of first antibiotics, d	6 (1–33)	10 (2–33)	6 (1–13)	<0.001
Other complications (Clavien–Dindo ≥2)	19 (16.5)	5 (22.7%)	14 (15.1%)	0.399
Length of hospital stay, d	13 (1–90)	29 (12–77)	11 (1–90)	<0.001

*Note*: Data are expressed as median (range) or *n* (%).

Abbreviations: ASA‐PS, the American Society of Anesthesiologists physical status; CRP, C‐reactive protein; eGFR, estimated glomerular filtration rate; NSAIDs, nonsteroidal antiinflammatory drugs; WBC, white blood cell.

^a^ Fisher's exact test was used. Other comparisons were performed using Pearson's chi‐square test for categorical variables.

^b^ The data about irrigation volume is missing in 26 patients; each group includes 18 and 71 patients, respectively.

The abscess group had higher age (*p* = 0.003), more female gender (*p* = 0.044), higher rates of diabetes (*p* = 0.027), current use of nonsteroidal antiinflammatory drugs (NSAIDs) (*p* = 0.015), ongoing cancer chemotherapy (*p* = 0.024), and higher American Society of Anesthesiologists physical status (ASA‐PS) (*p* = 0.047). Lower eGFR (*p* < 0.001) and higher C‐reactive protein (CRP) (*p* = 0.013) were also observed in the abscess group. The variables were comparable for intraoperative factors. Regarding operative outcomes, the abscess group had delayed oral intake (*p* < 0.001), longer antibiotic administration (*p* < 0.001), and more extended hospital stay (*p* < 0.001).

### 3.2 Risk factors for residual abscess

Ten variables, including preoperative and operative factors, were analyzed using the multiple logistic regression analysis (Table 2). While the univariable analyses revealed that age ≥75 y, ASA‐PS ≥3, diabetes, NSAIDs use, ongoing chemotherapy, ASA‐PS ≥3, time from onset to operation ≥72 h, on‐admission eGFR <50, on‐admission CRP ≥20, operative time >2 h were potential predictors for abscess formation, the multivariable analysis identified NSAIDs use (OR 3.76, 95% CI 1.09–13.03; *p* = 0.037), chemotherapy (OR 13.56, 95% CI 2.18–84.4; *p* = 0.005), and eGFR<50 (OR 4.72, 95% CI 1.30–17.17; *p* = 0.018) as independent predictors.

**TABLE 2:** Multiple logistic regression analysis assessing risk factors for residual abscess (*n* = 115).

Variables	Number, yes	Univariable analysis		Multivariable analysis	
		OR (95%CI)	*p* value	OR (95%CI)	*p* value
Age ≥75 y	39	1.84 (0.71–4.74)	0.207		
Diabetes mellitus	14	3.98 (1.22–13.04)	0.022	2.63 (0.60–11.55)	0.202
NSAIDs	28	3.47 (1.30–9.29)	0.013	3.76 (1.09–13.03)	0.037
Cancer chemotherapy	7	6.67 (1.37–32.37)	0.019	13.56 (2.18–84.4)	0.005
Stage IV Cancer	8	2.78 (0.61–12.64)	0.207		
ASA‐PS ≥3	24	2.75 (0.99–7.64)	0.052	1.36 (0.40–4.68)	0.622
Time to operation ≥72 h	9	3.91 (0.96–16.01)	0.069	5.28 (0.80–34.64)	0.083
On‐admission eGFR <50 mL/min/1.73m^2^	30	4.80 (1.79–12.86)	0.002	4.72 (1.30–17.17)	0.018
On‐admission CRP ≥20 mg/dL	24	2.71 (0.98–7.54)	0.056	0.66 (0.16–2.65)	0.556
Operation time >2h	25	3.29 (1.20–9.00)	0.020	3.49 (0.95–12.89)	0.061

Abbreviations: ASA‐PS, the American Society of Anesthesiologists physical status; CRP, C‐reactive protein; eGFR, estimated glomerular filtration rate; NSAIDs, nonsteroidal antiinflammatory drugs; WBC, white blood cell.

### 3.3 Potential scoring model

**FIGURE 2:**
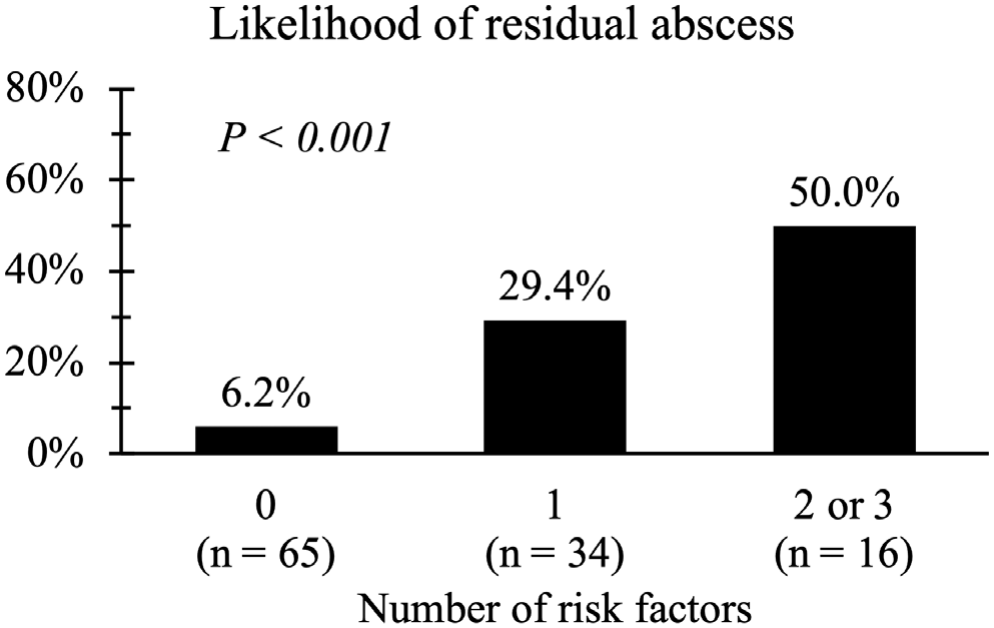
The association between the number of risk factors and residual abscess formation.

A scoring model was created using the above three predictors and the occurrence rate of residual abscess was compared between different scores depending on how many risks each patient had (0 vs. 1 vs. 2 and more). While the likelihood was very low when a patient had no risks (6.2%), the possibility increased as the number of risks increased (Figure 2). In other words, the number of risk factors correlates with the possibility of developing a residual abscess (*p* < 0.001).

### 3.4 Microbiological perspective

Ascites was submitted for culture in 90 patients (78.3%). The samples were obtained intraoperatively before surgical repair. Including 32 patients whose results were “no growth (negative)” and three patients whose results were “normal flora,” detailed information about microorganisms in the remaining patients is summarized in Table 3. The main pathogens isolated from the ascites culture were *Candida* (41.1%), *Streptococcus* (24.4%), *Escherichia coli* (5.6%), *Klebsiella* (5.6%), *Staphylococcus* (4.4%), *Enterococcus* (3.3%) *Pseudomonas* (3.3%), and *Enterobacter* (2.2%). When the relationship between microorganisms and abscess formation was examined, all three patients with *Enterococcus* (two with *Enterococcus faecium* and one with *faecalis*) in the ascites developed residual abscesses (100%). Susceptibility testing showed that the strains isolated in all three patients resisted cephalosporins, which were used as the first antibiotics.

**TABLE 3:** Detailed information regarding ascites culture (*n* = 90).

	Detection (*n* = 90)	Abscess formation, yes
No growth	35.6% (*n* = 32)	25.0%
*Streptococcus*	24.4% (*n* = 22)	22.7%
*Escherichia coli*	5.6% (*n* = 5)	20.0%
*Klebsiella*	5.6% (n = 5)	20.0%
*Staphylococcus*	4.4% (*n* = 4)	25.0%
*Enterococcus*	3.3% (*n* = 3)	100.0%
*Pseudomonas*	3.3% (*n* = 3)	33.0%
Normal flora	3.3% (*n* = 3)	33.0%
*Enterobacter*	2.2% (*n* = 2)	0%
*Candida*	41.1% (*n* = 37)	29.7%
*Aspergillus*	1.1% (*n* = 1)	0%
Others	18.9% (*n* = 17)	17.6%

### 3.5 Usefulness of postoperative blood test as a predictor

**FIGURE 3:**
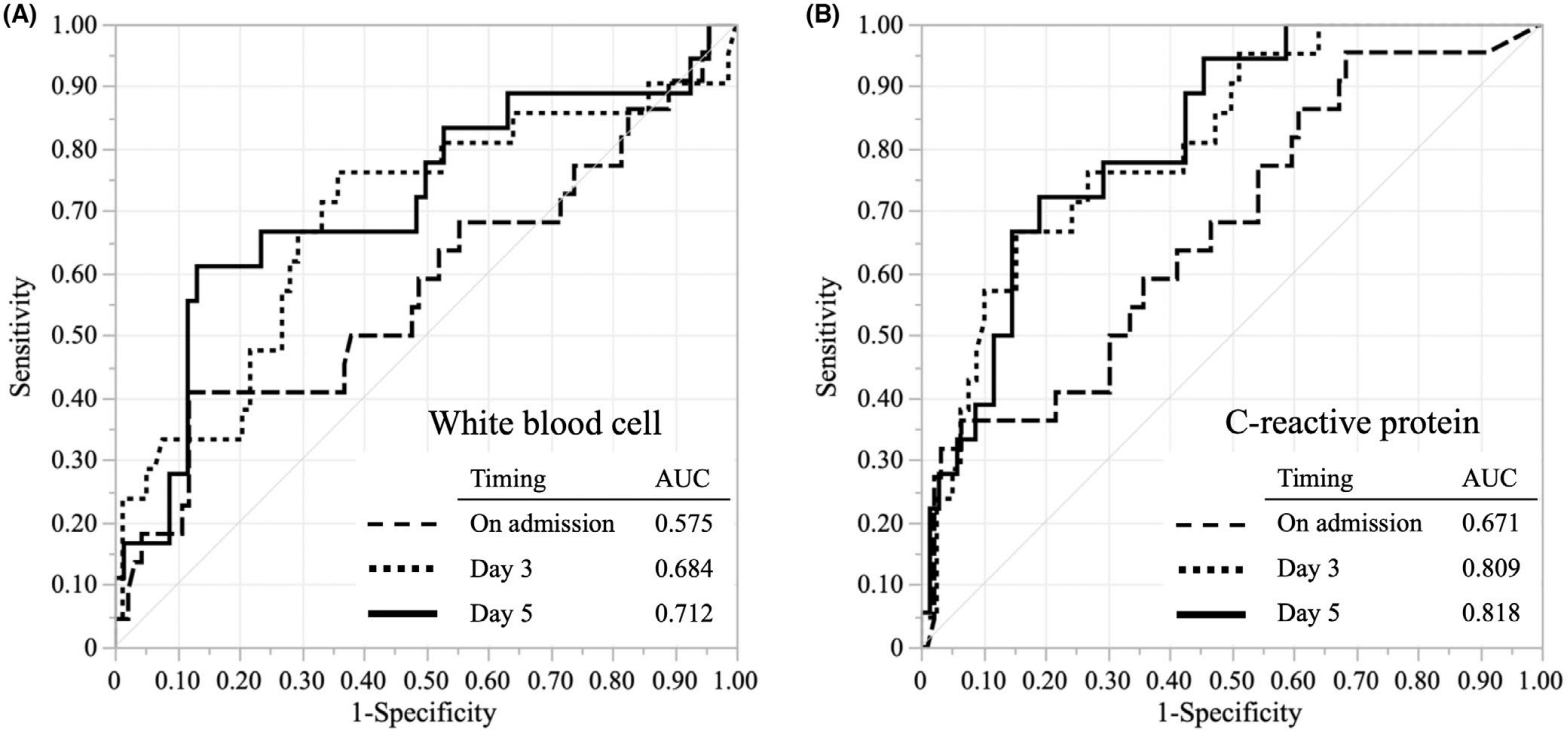
Comparison of the ability to predict abscess formation. (A) White blood cell. (B) C‐reactive protein. AUC, area under the curve.

The predictive abilities of white blood cell (WBC) count and CRP at different timepoints (on admission, d 3, and d 5) for residual abscesses were evaluated (Figure 3). Although both WBC and CRP had good predictive values, CRP on d 5 had the highest area under the curve of 0.818. A cutoff score of 10.0 mg/dL on d 5 (sensitivity: 72.2%, specificity: 80.9%, data not shown) was suggested by ROC curve analysis.

## 4 DISCUSSION

**FIGURE 4:**
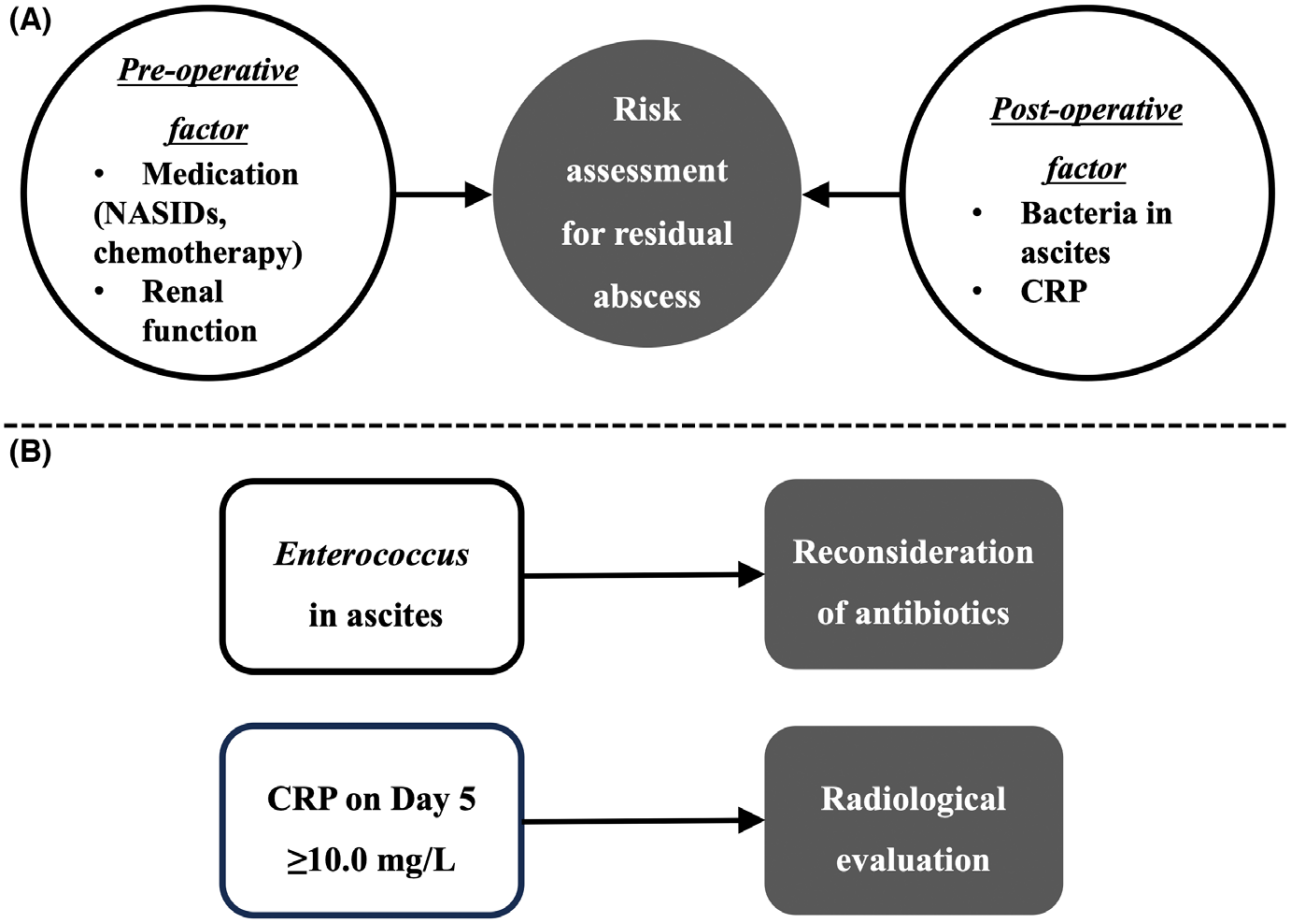
Suggestions for risk evaluation and treatment of residual abscess in gastroduodenal perforation. (A) Factors contributing to risk evaluation. (B) Factors contributing to early detection and treatment. CRP, C‐reactive protein; NSAIDs, nonsteroidal antiinflammatory drugs.

The present study demonstrated the potential risk factors for residual abscess formation after surgical treatment of GD perforation. Figure 4 summarizes the risk assessment diagram based on the newly found perspectives. Combining preoperative and postoperative information may be helpful in risk assessment (Figure 4A). Paying attention to ascites culture and inflammatory marker results would also contribute to prompt diagnosis and treatment (Figure 4B). In general, these factors are already well known to surgeons when assessing the risk of abdominal surgery. Still, it should be emphasized that a systematic assessment can be made by maximizing existing knowledge.

The incidence of residual abscess formation at our institution (19.1%) was higher than that of the National Clinical Database in Japan (8.0%).^2^ Since this reference did not provide detailed patient characteristics, a fair comparison using the identified risk factors is impossible. However, the median length of hospital stay (open surgery; 17 vs. 20–21 d, laparoscopic surgery; 13 vs. 13–14 d) was comparable and the incidence of reoperation (3.5% vs. 6.1%), the rates of CD ≥3 complications (13% (15/115) vs. 19.8%) and 30‐d mortality (1.7% (2/115) vs. 6.8%) were lower in our institution, suggesting that the surgical indication itself was not wrong. We anticipate that the incidence may decrease after the new findings from the current study are shared with our team.

As we assume that delayed wound healing and severe preoperative contamination are responsible for abscess formation, their potential relationship with the identified risk factors should be evaluated. First, NSAIDs may prevent tissue damage during healing. Unfortunately, we don't have detailed data about the duration of prescriptions and the reasons for their use in this cohort. However, long‐term use of NSAIDs causes damage to the GD mucosa through several mechanisms, including impairment of mucosal barrier properties, suppression of gastric prostaglandin synthesis, reduction of gastric mucosal blood flow, and interference with the repair of superficial injury.^6^ As a result, the healing process after surgical suturing may also take time. Second, anticancer drugs have been reported to delay wound healing. Anti‐vascular endothelial growth factor (VEGF) agents and tyrosine kinase inhibitors specifically target angiogenesis, which is associated with impaired wound healing and gastric perforation.^7^, ^8^, ^9^ For this reason, they are recommended to be discontinued before surgery or in the event of impaired wound healing and gastric perforation.^10^, ^11^ In this cohort, three out of seven patients were treated with anti‐VEGF agents, and two developed a residual abscess (Table S1). Third, as renal dysfunction is a known factor contributing to the severity and prognosis of various infections such as pneumonia and urinary tract infections,^12^, ^13^, ^14^, ^15^ it is natural to assume that it would also negatively affect the outcome of intraabdominal infection. In this study, 26% (30 of 115) of patients had moderate renal dysfunction (eGFR <50) on admission. While the exact cause of renal dysfunction remains unclear, a plausible explanation is preexisting chronic kidney disease (CKD). Although we could not obtain precise data on preexisting CKD in the study population (only five patients had chronic renal failure before the visit based on medical records), the prevalence of hypertension (76.7% vs. 18.3%, *p* < 0.001) and diabetes (23.3% vs. 8.5%, *p* = 0.036), the leading causes of CKD, was higher in patients with renal dysfunction. Another possible explanation is that the time between the onset of GD perforation and surgery affected the patients' renal function. Since 48.7% (56 of 115) of the patients presented to the hospital 48 h after the onset of symptoms, it is conceivable that the systemic inflammation had progressed considerably before they were admitted to the hospitals and, as a result, several patients had already developed renal dysfunction on admission due to dehydration. Notably, patients with renal dysfunction had significantly higher median CRP levels on admission than those without renal dysfunction (13.5 mg/dL vs. 1.2 mg/dL, *p* < 0.001).

The results of the postoperative tests are also worth mentioning. Interestingly, *Enterococcus* in ascites was strongly associated with residual abscess formation. Although we selected broad‐spectrum antibiotics before surgery, most antibiotics would not cover *Enterococcus* because 97% of patients (112 of 115) received cephalosporins or penicillin/ampicillin as antibiotic agents on admission. *Enterococcus* species are intrinsically resistant to several antimicrobials, including cephalosporins and trimethoprim‐sulfamethoxazole, and exhibit low levels of resistance to beta‐lactams and aminoglycosides.^16^ They are also less susceptible to penicillin/ampicillin and other β‐lactam antibiotics than other Gram‐positive coccus because they have low‐affinity penicillin‐binding proteins. *Enterococcus* are divided into *Enterococcus faecium* and *Enterococcus faecalis*. Penicillin/ampicillin resistance is more common in *Enterococcus faecium* than in *Enterococcus faecalis*.^17^, ^18^ Although the detection frequency was not high, only 4.3%, it means that antibiotics must be changed immediately if *Enterococcus* is suspected by Gram stain or culture. In this sense, ascites culture would be very informative; therefore, samples should be sent for culture in all cases.

In addition to appropriate antibiotic selection, radiologic evaluation for prompt diagnosis is also essential. Based on our data, CT should be performed around d 5 in case of suspicion of residual abscess if the CRP is above 10.0 mg/dL. Since CT performed in the very acute phase (d 1 to d 3) usually contains many nonspecific postoperative changes, this timing is reasonable. We will prospectively investigate the feasibility of this parameter in our department.

This study has some limitations. First, this study only focused on risk assessment and did not guide how to prevent residual abscess formation. Although improved recovery programs have been reported to reduce their incidence,^19^ further research on more detailed surgical techniques and more specific perioperative management is needed. Second, our database lacked information on *Helicobacter pylori* infection because the diagnosis was made at other institutions after discharge in many cases. As it's a major cause of GD perforations, its impact on residual abscesses should be assessed in future studies. Finally, the power of the analysis may be weak due to the relatively small number of patients. However, as this is the first exploratory study to propose a risk stratification strategy, we hope this study will allow this topic to receive more attention.

In conclusion, since the pathogenesis of residual abscess is likely to be multifactorial, careful evaluation of potential risk factors from all angles will help us to provide prompt and precise treatment to patients, including medication history, renal function, microorganisms, and inflammatory biomarkers.

## AUTHOR CONTRIBUTIONS

K.I. and S.Y. designed the study. K.I. collected the data and wrote the article. K.I., S.Y., T.Ku., K.T., S.Hon., S.Hos., and T.M. processed the data. T.Ka., S.N., and T.H. critically reviewed and proofread the article. All authors contributed to the article and approved the submitted version.

## CONFLICT OF INTEREST STATEMENT

The authors of this article have no conflicts of interest to disclose as described by the *Annals of Gastroenterological Surgery*.

## ETHICS STATEMENT

Approval of the research protocol: The protocol for this research project was approved by Kobe City Medical Center West Hospital Ethics Board (Approval no. 23‐023).

Informed Consent: N/A.

Registry and the Registration No. of the study/Trial: N/A.

Animal Studies: N/A.

## Supporting information


**Table S1.** The association between anticancer‐drugs and residual abscess.

## Data Availability

The data supporting the findings of this study are available from the corresponding author upon reasonable request.

## References

[1] Treuheit J , Krautz C , Weber GF , Grützmann R , Brunner M . Risk factors for postoperative morbidity, suture insufficiency, Re‐surgery and mortality in patients with gastroduodenal perforation. J Clin Med. 2023;12(19):6300. 37834943 10.3390/jcm12196300 PMC10573308

[2] Hoshino N , Endo H , Hida K , Kumamaru H , Hasegawa H , Ishigame T , et al. Laparoscopic surgery for acute diffuse peritonitis due to gastrointestinal perforation: a Nationwide epidemiologic study using the National Clinical Database. Ann Gastroenterol Surg. 2022;6(3):430–444. 35634193 10.1002/ags3.12533 PMC9130886

[3] Kobayashi T , Tabuchi S , Koganezawa I , Nakagawa M , Yokozuka K , Ochiai S , et al. Early identification of patients with potential failure of nonoperative management for gastroduodenal peptic ulcer perforation. Dig Surg. 2024;41(1):24–29. 38008080 10.1159/000535520

[4] Matsuo S , Imai E , Horio M , Yasuda Y , Tomita K , Nitta K , et al. Revised equations for estimated GFR from serum creatinine in Japan. Am J Kidney Dis. 2009;53(6):982–992. 19339088 10.1053/j.ajkd.2008.12.034

[5] Dindo D , Demartines N , Clavien PA . Classification of surgical complications: a new proposal with evaluation in a cohort of 6336 patients and results of a survey. Ann Surg. 2004;240(2):205–213. 15273542 10.1097/01.sla.0000133083.54934.ae PMC1360123

[6] Wallace JL . How do NSAIDs cause ulcer disease? Baillieres Best Pract Res Clin Gastroenterol. 2000;14(1):147–159. 10749095 10.1053/bega.1999.0065

[7] Nakamura H , Yokoyama Y , Uehara K , Kokuryo T , Yamaguchi J , Tsuzuki T , et al. The effects of bevacizumab on intestinal anastomotic healing in rabbits. Surg Today. 2016;46(12):1456–1463. 27172973 10.1007/s00595-016-1342-4

[8] Xue Y , Zhao F . Effect of bevacizumab in combination with chemotherapy for ovarian cancer on wound healing in patients: a meta‐analysis. Int Wound J. 2024;21(4):e14531. 38151891 10.1111/iwj.14531 PMC10961034

[9] Shah DR , Dholakia S , Shah RR . Effect of tyrosine kinase inhibitors on wound healing and tissue repair: implications for surgery in cancer patients. Drug Saf. 2014;37(3):135–149. 24526268 10.1007/s40264-014-0139-x

[10] Chen HX , Cleck JN . Adverse effects of anticancer agents that target the VEGF pathway. Nat Rev Clin Oncol. 2009;6(8):465–477. 19581909 10.1038/nrclinonc.2009.94

[11] Bose D , Meric‐Bernstam F , Hofstetter W , Reardon DA , Flaherty KT , Ellis LM . Vascular endothelial growth factor targeted therapy in the perioperative setting: implications for patient care. Lancet Oncol. 2010;11(4):373–382. 20171141 10.1016/S1470-2045(09)70341-9

[12] Ishigami J , Grams ME , Chang AR , Carrero JJ , Coresh J , Matsushita K . CKD and risk for hospitalization with infection: the atherosclerosis risk in communities (ARIC) study. Am J Kidney Dis. 2017;69(6):752–761. 27884474 10.1053/j.ajkd.2016.09.018 PMC5438909

[13] Sato R , Matsuzawa Y , Ogawa H , Kimura K , Tsuboi N , Yokoo T , et al. Chronic kidney disease and clinical outcomes in patients with COVID‐19 in Japan. Clin Exp Nephrol. 2022;26(10):974–981. 35657437 10.1007/s10157-022-02240-x PMC9164570

[14] James MT , Quan H , Tonelli M , Manns BJ , Faris P , Laupland KB , et al. CKD and risk of hospitalization and death with pneumonia. Am J Kidney Dis. 2009;54(1):24–32. 19447535 10.1053/j.ajkd.2009.04.005

[15] Wang HE , Gamboa C , Warnock DG , Muntner P . Chronic kidney disease and risk of death from infection. Am J Nephrol. 2011;34(4):330–336. 21860228 10.1159/000330673 PMC3169360

[16] Zaheer R , Cook SR , Barbieri R , Goji N , Cameron A , Petkau A , et al. Surveillance of enterococcus spp. reveals distinct species and antimicrobial resistance diversity across a one‐health continuum. Sci Rep. 2020;10(1):3937. 32127598 10.1038/s41598-020-61002-5 PMC7054549

[17] Cattoir V , Giard JC . Antibiotic resistance in Enterococcus faecium clinical isolates. Expert Rev Anti‐Infect Ther. 2014;12(2):239–248. 24392717 10.1586/14787210.2014.870886

[18] Depardieu F , Podglajen I , Leclercq R , Collatz E , Courvalin P . Modes and modulations of antibiotic resistance gene expression. Clin Microbiol Rev. 2007;20(1):79–114. 17223624 10.1128/CMR.00015-06 PMC1797629

[19] Tandup C , Chauhan A , Chauhan R , Thakur V , Sahu S , Kaman L , et al. Impact of tailored‐enhanced recovery after surgery versus conventional care in patients of gastro‐duodenal perforation: a pilot randomized control trial. Cureus. 2023;15(9):e45349. 37849602 10.7759/cureus.45349 PMC10578038

