# Supplementary material for: Risk stratification of residual abscess after surgical treatment for gastroduodenal perforation
Source: Ann Gastroenterol Surg. 2024 Nov 4;9(2):263–70. doi: 10.1002/ags3.12877 (PMC11877338; doi:10.1002/ags3.12877)
Supplement: Supplementary file 1 — Table S1. The association between anticancer‐drugs and residual abscess. [file AGS3-9-263-s001.docx]

Supplementary Table. The association between anticancer-drugs and residual abscess.

| Patient No. | Diagnosis | Anti-cancer drugs | Residual abscess |
| --- | --- | --- | --- |
| 1 | Gastric cancer | Paclitaxel + Ramucirumab（anti-VEGF） | Yes |
| 2 | Gastric cancer | TS-1 + CDDP | Yes |
| 3 | Metastasis of colon cancer | FOLFOXILI + Bevacizumab（anti-VEGF） | No |
| 4 | Metastasis of colon cancer | Iriotecan + TS-1 + Bevacizumab（anti-VEGF） | Yes |
| 5 | Breast cancer | Fulvestrant + Abemaciclib | No |
| 6 | Breast cancer | Anastrozole | No |
| 7 | Lung cancer | CDDP + VP-16 | Yes |
